# Is fibroblast growth factor 23 a new cardiovascular risk marker in gestational diabetes?

**DOI:** 10.1590/2359-3997000000287

**Published:** 2017-09-04

**Authors:** Muhammed Kizilgul, Seyfullah Kan, Selvihan Beysel, Mahmut Apaydin, Ozgur Ozcelik, Mustafa Caliskan, Mustafa Ozbek, Seyda Ozdemir, Erman Cakal

**Affiliations:** 1 Department of Endocrinology and Metabolism Diskapi Teaching and Research Hospital Ankara Turkey Department of Endocrinology and Metabolism, Diskapi Teaching and Research Hospital, Ankara, Turkey; 2 Schulze Diabetes Institute Department of Surgery University of Minnesota Minneapolis Minnesota USA Schulze Diabetes Institute, Department of Surgery, University of Minnesota, Minneapolis, Minnesota, USA; 3 Department of Biochemistry Diskapi Teaching and Research Hospital Ankara Turkey Department of Biochemistry, Diskapi Teaching and Research Hospital, Ankara, Turkey

**Keywords:** Gestational diabetes mellitus, fibroblast growth factor 23, cardiovascular risk

## Abstract

**Objective:**

This study was designed to compare the serum levels of fibroblast growth factor 23 (FGF23) among patients with gestational diabetes mellitus (GDM) and healthy pregnant women, and to evaluate the association between hormonal and metabolic parameters.

**Subjects and methods:**

A total of 82 pregnant women were consecutively enrolled in the study. Of these, 46 were diagnosed as having GDM; the remaining 36 healthy pregnant women served as controls in a cross-sectional study design. The womens’ ages ranged from 22 to 38 years and gestational ages, from 24 to 28 weeks. Serum samples were analyzed for FGF23 levels using an enzyme-linked immunosorbent assay.

**Results:**

Serum FGF23 levels were increased in patients with GDM compared with controls (median, 65.3 for patients with GDM vs. 36.6 ng/mL for healthy controls; p = 0.019). Mean fasting glucose (105.6 ± 7.4 vs. 70.2 ± 7.2 mg/dL, p < 0.001), HbA1c (5.6 ± 0.5 vs. 4.9 ± 0.5%, p < 0.001), insulin (median, 11.1 vs. 8.7 µIU/mL, p = 0.006) and HOMA-IR (3.0 (1.8) vs 1.4 (0.6), p < 0.001) levels were significantly higher in patients with GDM than in controls. Serum FGF23 level was positively correlated with body mass index (r^2^ = 0.346, p < 0.05), FPG (r^2^ = 0.264, p < 0.05), insulin (r^2^ = 0.388, p < 0.05), HOMA-IR (r^2^ = 0.384, p < 0.05).

**Conclusion:**

Serum FGF23 levels were higher in women with GDM compared with controls. The present findings suggest that FGF23 could be a useful marker of cardiovascular disease in GDM.

## INTRODUCTION

Gestational diabetes mellitus (GDM) is characterized by glucose intolerance with onset or first recognition during pregnancy. GDM is one of the most commonly encountered complications of pregnancy, affecting 1.1-14.3% of pregnant women (1). GDM poses an increased risk of adverse maternal and fetal outcomes (2). The disease has significant health implications for both mother and child, including the development of type 2 diabetes mellitus (T2DM), obesity, and even cardiovascular disease later in life (3-5).

Fibroblast growth factors (FGF) play a role in various biologic activities such as angiogenesis, mitogenesis, cell differentiation, cell migration, and the repair of injured tissue (6). Fibroblast growth factor 23 (FGF23) is a 32 kDa (251 amino acids) polypeptide with an N-terminal and C-terminal region that is released by osteocytes and osteoblasts in response to elevated serum phosphorus levels (7). FGF23 is a hormone involved in phosphorus homeostasis, vitamin D metabolism, and bone mineralization. FGF23 is included in the group of hormones called FGFs, along with FGF19 and FGF21. The heparin-binding region of FGF23 differs from the topologic point of view, unlike many other FGFs that attach to heparin sulfate in the extracellular matrix exerting endocrine influences. Accordingly, FGF23 binds less vigorously to the extracellular matrix, hence it is more likely to enter the systemic circulation, which allows FGF23 to present paracrine and autocrine effects (8,9). Higher FGF23 levels, even in individuals without renal insufficiency, correspond to an increased risk of cardiovascular mortality in the normal population (10), and cardiovascular risk factors including vascular dysfunction, atherosclerosis, and left ventricular hypertrophy (11-13). GDM is associated with an increased risk of T2DM and cardiovascular disease. This study was designed to compare serum FGF23 levels of GDM women with those of non-GDM women, and to evaluate the association between hormonal and metabolic parameters.

## SUBJECTS AND METHODS

### Study population

A total of 82 pregnant women who were followed up by the Endocrinology and Metabolism clinic of Ankara Diskapi Teaching and Research Hospital were consecutively enrolled in the study. Of these, 46 women were diagnosed as having GDM; the remaining 36 healthy pregnant women served as controls in a cross-sectional study design. Ethics committee approval was obtained and written informed consent was given by the participants before the performance of any study procedures. The womens’ ages ranged from 22 to 38 years and gestational ages, from 24 to 28 weeks. Gestational age was estimated according to the date of the last menstrual period and simultaneous clinical evaluation (14).

International Association of Diabetes and Pregnancy Study Groups (IADPSG) criteria were used for the diagnosis of GDM. A 2-hour, 75-gr oral glucose tolerance test (OGTT) was performed on all pregnant women, at 24 to 28 weeks of gestation. Glucose levels after fasting, and 1 and 2 h after glucose administration < 92 mg/dL, < 180 mg/dL, and < 153 mg/dL, respectively, were considered normal; if the glucose level was higher than the standard at any point, the patient was diagnosed as having GDM (15).

Pregnant women with a thyroid disorder, infectious disease, hypertension, pre-eclampsia, hepatic or renal dysfunction, cardiac disease, metabolic bone disease, and fetal anomalies were excluded. The patients with GDM received several treatments (diet or diet plus insulin therapy) for maintaining blood glucose control. All blood samples were taken before starting treatment.

### Clinical, biochemical, and hormone measurements

Weight, height, systolic and diastolic blood pressure (BP) were measured. Body mass index (BMI) was calculated as weight (kg)/height (m)^2^. A venous blood sample was collected after an overnight fast of at least 8 hours. Samples were centrifuged within 30 to 45 minutes of collection and stored at -80°C. Insulin resistance was calculated using homeostasis model assessment (HOMA-IR) (16).

Plasma glucose was determined using the glucose oxidase method (Siemens ADVIA 2400 Chemistry System, Siemens Medical Solutions Diagnostics Tarrytown, NY, USA). The level of total cholesterol was determined using an enzymatic method (Siemens, ADVIA 2400 Chemistry System, Siemens Medical Solutions Diagnostics Tarrytown, NY, USA). Serum triglyceride was determined using the Trinder method without a blank serum (Siemens ADVIA 2400 Chemistry System, Tarrytown, NY, USA). Low-density lipoprotein cholesterol (LDL-C) and high-density lipoprotein cholesterol (HDL-C) were measured using the elimination/catalase method (Siemens ADVIA 2400 Chemistry System, Tarrytown, NY, USA). High-sensitivity C-reactive protein (HsCRP) was determined using the latex-enhanced immunoturbidimetric method (Siemens ADVIA 2400 Chemistry System, Tarrytown, NY, USA).

Thyroid-stimulating hormone (TSH) and insulin were measured using chemiluminescence immunoassays (Advia Centaur XP, Siemens Healthcare Diagnostics, Tarrytown, NY, USA).

### Measurement of FGF23

Serum samples were analyzed for FGF23 levels using an enzyme-linked immunosorbent assay (ELISA) (Aviscera Bioscience, Santa Clara, USA). According to manufacturer’s indications, the calculated overall intra-assay coefficient of variation (CV) was between 6.0 and 8.0% and the inter-assay CV was between 8.0 and 12.0%. The minimum detectable level of FGF23 was typical at ~15 pg/mL.

### Statistical analyses

Statistical analysis was performed using SPSS 18.0 (SPSS, Inc) software. Variables are presented as mean ± standard deviation (SD). Normality was tested using the Kolmogorov-Smirnov and Shapiro-Wilk *W* test. Student’s *t*-test was used for normally distributed continuous variables. The Mann-Whitney *U* test was used for continuous variables that were not normally distributed. Correlations were analyzed using Pearson and Spearman’s correlation. Statistical significance was defined as a p < 0.05.

## RESULTS

**Figure 1:**
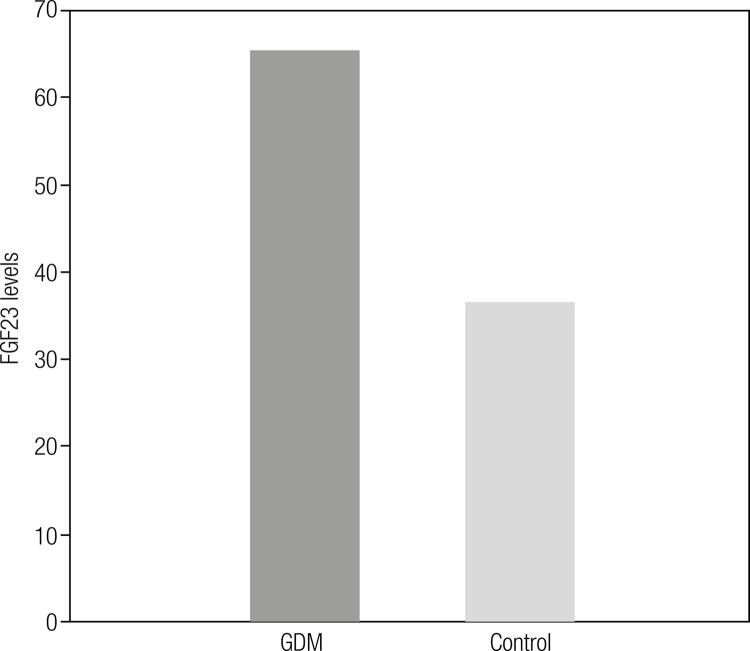
Plasma levels of FGF23 in the study groups.

The mean age (30.2 ± 4.8 vs. 29 ± 4.0 years, p = 0.278) was similar between the groups. Women with GDM had a significant higher body weight (78.2 ± 11.4 vs. 67.8 ± 12.2 kg, p = 0.001) and BMI (30.8 ± 4.6 vs. 26.9 ± 5.4 kg/m^2^, p = 0.002) as compared with the controls. The mean fasting glucose (105.6 ± 7.4 vs. 70.2 ± 7.2 mg/dL, p < 0.001), glycated hemoglobin (HbA1c) (5.6 ± 0.5 vs. 4.9 ± 0.5%, p < 0.001), insulin (11.1 (6.8) vs. 8.7 (2.6) µIU/mL, p = 0.006), and HOMA-IR (3.0 (1.8) vs. 1.4 (0.6), p < 0.001) levels were significantly higher in women with GDM than in controls. The mean serum FGF23 level (65.3 (213.5) vs. 36.6 (50.3) ng/mL, p = 0.019) was significantly higher in women with GDM as compared with controls (Figure 1). Clinical and biochemical characteristics of the women with GDM and controls are shown in Table 1. There were no significant differences between women with GDM and controls in terms of gestational weeks, height, phosphate and 25-OH Vitamin D levels (p > 0.05). The serum fasting glucose level was positively correlated with age (r = 0.365, p = 0.001), BMI (r = 0.295, p = 0,007), HbA1c (r = 0.564, p = 0.001), insulin (r = 0.327, p = 0.007), and HOMA-IR (r = 0.234, p = 0.058). Serum FGF23 level was positively correlated with BMI (r^2^ = 0.346, p < 0.05), FPG (r^2^ = 0.264, p < 0.05), insulin (r^2^ = 0.388, p < 0.05), and HOMA-IR (r^2^ = 0.384, p < 0.05) (Table 2).

**Table 1:** The clinical and biochemical characteristics of the women with gestational diabetics and controls

Parameters	GDM group (n = 40)			Control group (n = 40)			p
Age	30.9	±	5.6	28.0	±	4.6	0.023
Gestational age (week)	25.9	±	1.6	26.4	±	1.5	0.175
Height	159.5	±	5.5	159.1	±	4.6	0.778
Weight	78.2	±	11.4	67.8	±	12.2	**< 0.001**
BMI	30.8	±	4.6	26.9	±	5.4	**0.002**
FPG	105.6	±	7.4	70.2	±	7.2	**< 0.001**
HbA1c	5.6	±	0.5	4.9	±	0.3	**< 0.001**
Insulin	11.1 (6.8)			8.7 (2.6)			**0.006**
Phosphate	3.1 ± 0.5			3.2 ± 0.6			0.664
FGF23	65.3 (213.5)			36.6 (50.3)			**0.019**

Variables that are not normally distributed such as insulin and FGF23 levels are presented as median (IQR) and other variables with normal distribution are represented as mean ± SD.

BMI: body mass index; FPG: fasting plasma glucose.

**Table 2:** The correlation between FGF-23 levels and clinical, biochemical and hormonal parameters in PCOS group

	GDM group	Control group
Age	-0.003	0.073
Gestational age	-0.016	0.305
Height	0.206	0.012
Weight	0.012	0.037
BMI	0.346*	0.033
FPG	0.264*	-0.119
HbA1c	0.106	0.265
Insulin	0.388*	-0.210
HOMA-IR	0.384*	-0.284

* p < 0.05.

In conclusion, our study shows that FGF23 levels are significantly higher in pregnant women with GDM compared with those in pregnant controls. FGF23 is a member of the FGF19 subfamily of endocrine FGFs. FGF23 is principally expressed by osteocytes and osteoblasts in bone. It is also expressed in salivary gland and stomach, and at much lower concentrations in other tissues such as skeletal muscle, brain, mammary gland, liver, and the heart (17). It is well documented that higher FGF23 levels are associated with increased arterial stiffness, total body atherosclerosis, left ventricular hypertrophy, and consequently, there is an increased risk cardiovascular mortality, even in patients without renal insufficiency. A recent meta-analysis of prospective cohort studies reported that higher FGF23 levels were associated with an elevated risk of all-cause mortality, cardiovascular disease events, cardiovascular mortality, stroke, and heart failure (18). The segregation of the FGF23 polymorphism is significantly related to elevated serum FGF23 levels and cardiac complications in children with Kawasaki disease (19). There are several mechanisms that suggest a role of FGF23 in cardiovascular disease. One possible mechanism is the involvment of FGF23 in the complex process of vascular calcification (20). 1,25(OH)_2_D_3_ is the primary regulator of FGF23 production via osteoblasts in bone, and increased FGF23 levels cause a reduction in 1,25(OH)2D3 levels (21). The decrease in 1,25(OH)_2_D_3_ can cause elevated angiotensin II production via an increase in renin expression, which results in hypertension and cardiac hypertrophy (22-24). The decrease in vitamin D levels is associated with adverse outcomes in the general population (25). FGF23 requires a cofactor known as α-klotho for activation of FGF signaling (26). Soluble Klotho protects the heart via inhibition of the transient receptor potential cation channel 6 (TRPC6) gene whose overexpression leads to cardiac hypertrophy and remodeling (27). Isakova and cols. proposed that elevated levels of FGF23 led to Klotho deficiency (28). Andrukhova and cols. reported that FGF23 increased renal sodium reabsorption, thus causing hypertension and cardiac hypertrophy (29). It has been reported that levels of FGF23 correlated with different inflammatory markers (30,31). There is increasing evidence to suggest an association between increased blood pressure and hypophosphatemia (32). Gudmundsdottir and cols. reported that low serum phosphate levels were associated with the development of hypertension (33). The hypophosphatemic effect of increased FGF23 could explain the association of the latter with increased cardiovascular mortality. The decrease in the level of 1,25(OH)_2_D_3_, reduction in expression of soluble Klotho, activation of the renin-angiotensin system, increase in sodium retention in the kidneys, increase in inflammatory markers, and hypophosphatemia could be explanations for the effect of FGF23 on the cardiovascular system. GDM contributes to vascular dysfunction, as recently reported in a meta-analysis (34). The hypothesis of whether FGF23 levels are high in patients with GDM, which brings increased risk of cardiovascular disease, was tested in our study. We found that FGF23 levels increased in patients with GDM, and the FGF23 level was correlated with BMI, FPG, insulin, and HOMA-IR. The findings of our study suggest that there may be other possible mechanisms that contribute to increased cardiovascular disease risk in patients with GDM, regardless of increased plasma glucose.

Some studies evaluated the association of FGF23 with insulin resistance and DM. Hypoglycemia and profoundly elevated peripheral insulin sensitivity were observed in the vitamin D signaling cascade in healthy FGF23-null mice (35). Ali and cols. reported FGF23 levels were directly correlated with HOMA-IR in obese adolescents (36). Wojcik and cols. observed an inverse correlation between FGF23 levels and HOMA-IR in obese adolescents (37). Holecki and cols. demonstrated elevated FGF23 levels were associated with inflammation, but not with obesity and insulin resistance (38). In our study, FGF23 was correlated with insulin resistance. A recent study reported that serum FGF23 was associated with bone mineral density and preclinical vascular disease in patients with T2DM and their findings suggested that influences of FGF23 in these patients might be different from the effects in other populations (39).

To our knowledge, the present study is the first to evaluate FGF23 levels in women with GDM. A relatively small sample size and being a single-center study are limitations of this study.

Taken together, we have shown evidence that maternal FGF23 levels are significantly increased in GDM, which might contribute to increased metabolic and cardiovascular risk in these patients. Furthermore, we have demonstrated that body mass index, fasting plasma glucose, and HOMA-IR are independently associated with serum FGF23 concentrations. The present findings suggest that FGF23 could be a useful marker of cardiovascular disease in GDM; however, comprehensive studies covering larger populations are needed to enlighten the relationship between FGF23 and GDM.
